# Different Array CGH profiles within hereditary breast cancer tumors associated to BRCA1 expression and overall survival

**DOI:** 10.1186/s12885-016-2261-x

**Published:** 2016-03-15

**Authors:** Carolina Alvarez, Andrés Aravena, Teresa Tapia, Ester Rozenblum, Luisa Solís, Alejandro Corvalán, Mauricio Camus, Manuel Alvarez, David Munroe, Alejandro Maass, Pilar Carvallo

**Affiliations:** Department of Cellular and Molecular Biology, Faculty of Biological Sciences, Pontificia Universidad Católica de Chile, Santiago, Chile; Mathomics, Center for Mathematical Modeling (UMI 2807 CNRS) and Center for Genome Regulation (Fondap 15090007), University of Chile, Santiago, Chile; Laboratory of Molecular Technology Advanced Technology Program, SAIC-Frederick, Inc., National Cancer Institute-Frederick, Frederick, MD USA; Department of Anatomo-Pathology, Faculty of Medicine, Pontificia Universidad Católica de Chile, Santiago, Chile; Cancer Center, Faculty of Medicine, Pontificia Universidad Católica de Chile, Santiago, Chile; Clinica Las Condes, Santiago, Chile; Department of Mathematical Engineering, University of Chile, Santiago, Chile; Department of Molecular Biology and Genetics, Faculty of Science, Istanbul University, Istanbul, 34134 Turkey

**Keywords:** Breast cancer, BRCAX, Array CGH, Tumor suppressor, Oncogenes, Genomic losses, Genomic gains

## Abstract

**Background:**

Array CGH analysis of breast tumors has contributed to the identification of different genomic profiles in these tumors. Loss of DNA repair by BRCA1 functional deficiency in breast cancer has been proposed as a relevant contribution to breast cancer progression for tumors with no germline mutation. Identifying the genomic alterations taking place in BRCA1 not expressing tumors will lead us to a better understanding of the cellular functions affected in this heterogeneous disease. Moreover, specific genomic alterations may contribute to the identification of potential therapeutic targets and offer a more personalized treatment to breast cancer patients.

**Methods:**

Forty seven tumors from hereditary breast cancer cases, previously analyzed for BRCA1 expression, and screened for germline *BRCA1* and *2* mutations, were analyzed by Array based Comparative Genomic Hybridization (aCGH) using Agilent 4x44K arrays. Overall survival was established for tumors in different clusters using Log-rank (Mantel-Cox) Test. Gene lists obtained from aCGH analysis were analyzed for Gene Ontology enrichment using GOrilla and DAVID tools.

**Results:**

Genomic profiling of the tumors showed specific alterations associated to *BRCA1* or *2* mutation status, and BRCA1 expression in the tumors, affecting relevant cellular processes. Similar cellular functions were found affected in BRCA1 not expressing and *BRCA1* or *2* mutated tumors. Hierarchical clustering classified hereditary breast tumors in four major, groups according to the type and amount of genomic alterations, showing one group with a significantly poor overall survival (*p* = 0.0221). Within this cluster, deletion of *PLEKHO1, GDF11, DARC, DAG1* and *CD63* may be associated to the worse outcome of the patients.

**Conclusions:**

These results support the fact that BRCA1 lack of expression in tumors should be used as a marker for BRCAness and to select these patients for synthetic lethality approaches such as treatment with PARP inhibitors. In addition, the identification of specific alterations in breast tumors associated with poor survival, immune response or with a BRCAness phenotype will allow the use of a more personalized treatment in these patients.

## Background

Breast cancer is the first cause of female death by neoplasm around the world. In Chile, mortality rate due to breast cancer is in first place with 15.5/100.000 women (DEIS, MINSAL 2011). As all cancers, it has been described that breast cancer is driven by several alterations in tumor suppressor genes and oncogenes. Within these alterations, somatic mutations [1], gene deletion or duplication, and promoter hypermethylation [2] are described as the most frequent mechanisms occurring in cancer, and contributing to neoplastic progression [3, 4]. Mutations or alterations in tumor suppressor genes such as gene or chromosomal deletions can be found at different frequencies between tumors, being possible to find a cancer driver alteration in a low proportion of tumors [4]. Several methodologies, as next generation sequencing and array-CGH, are being used in order to detect and identify these mutations and rearrangements. Comparative genomic hybridization (CGH) and, more recently, array-based CGH have been extensively used in the analysis of gains and losses in tumor DNA [5, 6]. Among the most common genomic alterations described in sporadic and hereditary breast tumors are losses at chromosomes 8p, 11q, 13q and 17p; and gains within chromosomes 1q, 8q, 17q and 20q [7–12]. Through the years, several groups have intended to associate genomic alterations with different breast tumor characteristics. Regarding hereditary tumors, which are the focus of this study, the main findings relay on the association of genomic instability levels with the presence of BRCA1/2 abnormalities [8, 13, 14] or with immunohistochemical phenotypes [15]. In this sense, tumors with *BRCA1/2* mutations, *BRCA1* promoter hypermethylation/loss of expression, and “basal like” phenotype are shown to have higher instability. These findings are in coherence with BRCA1 and BRCA2 nuclear role in DNA repair, and support their relevance, not only for cancer predisposition, but also for cancer progression. These studies add important and valuable information to the field, nevertheless the complexity and genetic heterogeneity of breast cancer, and the genetic heterogeneity of worldwide populations, support the need of further studies expanding in the analysis of hereditary tumors.

Loss of BRCA1 expression has been described to be associated frequently to LOH [16] and promoter hypermethylation [13, 16, 17] in sporadic and hereditary cases. Few somatic mutations have been found recently for these genes. More recently, miRNA regulation of BRCA1 mRNA stability appears as a new mechanism contributing to BRCA1 silencing [18–20]. Interestingly, little has been done investigating genomic profiles in breast cancer tumors in association with BRCA1 expression. These studies have been mainly directed to triple negative sporadic breast cancer tumors [13, 21, 22].

The aim of the present work is to evaluate the genomic profiles of a Chilean subset of hereditary breast cancer tumors by array-CGH, highlighting the different alterations found in tumors with loss of BRCA1 expression, and in tumors with germline BRCA mutations. In addition, we identified hereditary tumors clusters in groups with different levels of genomic instability, and significant differences in overall survival. We identified particular genomic alterations in BRCA1 not expressing tumors relevant to functions associated with BRCA1/2 mutated tumors.

## Methods

### Patients and tumors

Families were previously selected from 1999 to 2004 from three health centers in Santiago, using standard criteria for hereditary breast cancer: 1) three women with breast cancer in at least two consecutive generations, 2) two women with breast cancer, one of them diagnosed before age of 41 and 3) at least one woman with breast and one with ovarian cancer [23]. All patients signed a written informed consent for the publication of clinical data and *BRCA1* and *BRCA2* mutational screening results. This protocol was approved by the Ethics Committee at the Faculty of Medicine, Pontificia Universidad Catolica de Chile. All patients were screened for *BRCA1* and *BRCA2* germline mutations as described by Gallardo et al [23]. A total of 47 formalin-fixed paraffin embedded (FFPE) tumor biopsies from surgically resected breast cancer tissue were collected from these patients. In this study, forty biopsies belong to BRCAX patients (hereditary cases with no *BRCA1*/*2* germline mutations), 3 to *BRCA1* patients and 4 to *BRCA2* patients.

### Immunohistochemistry

The histological type and grade of the tumors were classified according to the World Health Organization. Paraffin sections were processed for the detection of Estrogen Receptor (ER) and HER2 expression by immunohistochemistry at the Anatomo-Pathology department at clinical assessment. Briefly, 4 μm tumor sections were deparaffinized and re-hydrated prior to antigen unmasking with EDTA pH 8.0. Automated immunohistochemical staining was carried out using the *BioGenex* i *6000*™ Automated Staining System and the streptavidin–biotin complex (*sABC*) *peroxidase* method with DAB substrate (3, 3'- diaminobenzidine). Presence of ER and HER2 was evaluated using the following antibodies: anti-ER clone 6 F11 (1:40 dilution, Novocastra), and anti-HER2 clone CB11 (1:100 dilution, Novocastra). The interpretation of the slides was done in an independent manner by two pathologists. For ER and PR, positivity was scored as 1 % or more of the examined area positively stained, as established by the American Society of Clinical Oncology and the College of American Pathologists (ASCO/CAP). For HER2, scores 0 and 1+ indicate negativity and 2+ and 3+ positivity. In addition, we previously performed immunohistochemical detection of BRCA1 for our cohort of hereditary tumors [17].

### DNA extraction

Between 5000 and 10,000 tumor cells were manually microdissected from 5 μm Hematoxilin-Eosin (H&E) breast tumor sections, and collected into a sterile tube. DNA was extracted by Proteinase K digestion (0.4 mg/ml Proteinase K, 1 μM EDTA, 0.02 M Tris, 0.5 % Tween 20) for 48 h at 37 °C in a water bath under gentle shaking. After digestion, each DNA was precipitated with ethanol. In order to minimize the interference of polymorphic copy number variants (CNV), we prepared reference DNA from normal cells obtained from H&E sections of healthy lymph node biopsies from 6 of the analyzed *BRCAX* patients. Extracted DNA was quantified using a NanoDrop spectrophotometer (Thermo Fisher Scientific, DE).

### Array CGH

Ten to twenty nanograms of genomic DNA of each sample and reference were amplified with Phi29 DNA polymerase according to the supplier’s protocol (GenomiPhi, GE Healthcare). After verification of amplified product in a 0.8 % agarose gel we performed restriction digestion in order to obtain fragmented DNA of a suitable size for hybridization. All digestions were done with both AluI and RsaI for 4 h at 37 °C. Labeling reactions were performed with 6–8 μg of purified digested DNA using Bioprime CGH labeling kit (Invitrogen) according to the manufacturer’s instructions. The only variation was the extension of the labeling time to 18 h. Test DNA was labeled with Cy3-dUTP and reference DNA with Cy5-dUTP. Samples were then cleaned using MicroBioSpin6 Columns (BioRad) followed by ethanol precipitation. Specific activity of each fluorophore was estimated for all samples using a NanoDrop spectrophotometer (Thermo Fisher Scientific, DE). Equal amounts of test and reference labeled DNA (total volume of 50 μl) were mixed with 5 μg of Human Cot-1 DNA and 2X hybridization buffer (dextran sulfate 10 %, 3X SSC and Tween 20 1.5 %). Samples were hybridized under rotation for 40 h at 65 °C using a hybridization oven. Arrays were washed according to supplier’s protocol (Agilent Technologies).

### Oligonucleotide microarray platform

We used the Agilent oligonucleotide 4x44K microarrays for the array-based CGH analyses. This platform is based on the UCSC hg18 human genome (NCBI Build 36) and consists of 45,000 probes mainly directed to codifying sequences. All probes are 60mer oligonucleotides with an average spatial resolution of 43 Kb.

### Analyses

The hybridized microarrays were scanned with a GenePix 4100A scanner (Molecular Devices) and signal processing was done with either Feature Extraction software (Agilent Technologies) or GenePix Pro (Molecular Devices). Raw data was normalized using R package CGHnormaliter from Bioconductor (http://www.bioconductor.org/packages/2.6/bioc/html/CGHnormaliter.html). Deletions and gains were identified with DNA Analytics (Genomic Workbench, Agilent Technologies) using the ADM-1 (Aberration Detection Method-1) algorithm with a log2 ratio filter of 0.2, and a threshold of 4.0.

### Availability of data

The dataset supporting the conclusions of this articles is available in the Gene Expression Omnibus repository (http://www.ncbi.nlm.nih.gov/geo, accession number GSE70541)

### Hierarchical clustering

Using aberrations called by DNA Analytics we clustered our samples using R ‘hclust’ function with complete linkage. Every probe in each sample was represented by a nominal variable taking one of three values: loss, unaltered or gain. Then we used Hamming distance to compare samples, that is, we counted the number of probes in which two samples disagree. To avoid false positives induced by noise, we only considered probes that where altered on three or more samples. We examined the resulting hierarchical clustering and we found that the most informative partition was the one in four disjoint groups with similar size. We performed overall survival analysis to 10 years before census using Log-rank (Mantel-Cox) Test considering data available from all patients. Statistical significance was considered with a *p* value <0.05.

### Genomic instability of the tumors

For each tumor, total number of losses and gains were determined based upon called aberrations breakpoints identified by ADM-1. Using Student *t*-test we compared the genomic instability among the four clusters: Blue, Yellow, Green and Purple.

### Gene Ontology analyses

We performed ontological analyses with Gorilla [24] and DAVID [25] tools using gene lists obtained from the array-CGH analysis for different hereditary tumor groups: *BRCA1* or *BRCA2* mutated, BRCA1 not expressing, BRCA1 expressing, and clusters.

## Results

We analyzed 47 hereditary breast cancer tumors by array-CGH and found different alterations in relation to *BRCA1* and *BRCA2* mutation status, and to BRCA1 protein expression.

**Fig. 1:**
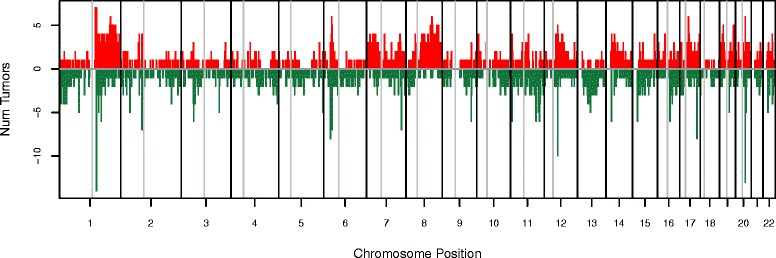
Graphical representation of the observed frequencies for gains (*red*) and losses (*green*) in hereditary tumors across all chromosomes. Frequencies are represented as number of tumors. Chromosomes are separated by thick black vertical lines, and centromeres are indicated with a thin grey vertical line

Tumor features and receptors status are specified in Table 1. Figure 1 shows a graphical representation of all probes involved in gained or lost regions across all chromosomes, and the number of tumors carrying such alterations; we observed that compared to gains, a greater number of deletions are present in unique tumors revealing heterogeneity at this level.

**Table 1:** Hereditary tumors, histopathological features and cancer family history

Tumor ID	Histological type	Tumor grade	IHC				Mutation detected		Family History
			ER	PR	HER2	BRCA1	BRCA1	BRCA2	
T6	IDC	III	-	-	-	-			4 breast, 1 esophageal cancer
T10	IDC	III	-	-	-	-			2 breast, 1 prostate cancer
T11	IDC	III	-	-	-	-			1 breast bilateral with ovarian cancer
T12	IDC	II	-	+	-	-			2 breast OR 1 breast, 1 ovarian, 1 stomach cancer
T17	IDC	III	-	-	-	-			2 breast, 1 uterine, 1 testicular cancer
T20	LCIS	_	-	-	1+	-			3 breast, 1 stomach cancer
T24	IDC	II	-	-	-	-	*YES*		1 bilateral and 2 breast, 1 gallbladder cancer, 1 melanoma
T39	IDC	III	-	-	-	-			1 bilateral and 3 breast cancer, 1 uterine, 1 stomach cancer
T41	IDC	III	-	-	-	-			4 breast, 2 stomach,1 prostate cancer
T42	IDC	III	-	-	-	-			4 breast, 2 stomach,1 prostate cancer
T43	IDC	III	-	-	-	-			4 breast, 2 stomach,1 prostate cancer
T45	IDC	II	-	+	-	-			1 bilateral breast, 4 breast, 1 testicular cancer
T25	IDC	III	+	+	-	-	*YES*		1 bilateral and 2 breast, 1 gallbladder cancer, 1 melanoma
T1	IDC	III	+	-	1+	-			5 breast, 1 stomach, 1 gallbladder, 1 other cancer
T3	IDC	III	+	+	-	-			2 breast, 1 uterine, 1 gallbladder, 1 esophageal cancer, 2 other cancer
T26	IDC	III	+	+	-	-			6 breast, 1 stomach cancer, 1 leukemia
T29	IDC	II	+	+	1+	-			5 breast, 1 liver, 2 stomach cancer
T32	IDC	I	+	+	-	-			3 breast, 1 prostate, 1 uterine cancer
T35	IDC	I	+	+	-	-			5 breast, 1 bilateral breast, 1 stomach, 1 pancreatic cancer
T36	IDC	I	+	+	-	-			3 breast cancer
T37	IDC	II	+	+	-	-			4 breast (1 bilateral), 1 testicular, 1 other cancer
T9	LCIS	_	+	+	3+	-			4 breast (1breast/colon), 3 stomach, 2 prostate, 1 pancreatic cancer
T15	IDC	II	+	+	2+	-			3 breast cancer, one in a male
T21	ILC	_	+	+	2+	-			3 breast, 1 stomach, 1 other cancer
T4	IDC	I	-	+	-	+			3 breast cancer
T5	IDC	III	-	-	-	+		*YES*	2 breast, 1 stomach cancer, 1 leukemia
T16	IDC	I	-	+	-	+			2 breast, 1 uterine, 1 stomach cancer
T23	IDC	III	-	-	-	+			1 breast, 1 prostate OR 1 breast, 1 stomach, 1 other cancer
T19	LCIS	_	-	+	2+	+			3 breast, 1 stomach cancer
T46	IDC	III	-	-	3+	+			1 bilateral and 1 breast cancer
T44	DCIS	_	+	+	-	+		*YES*	1 bilateral and 3 breast cancer, 1 ovarian cancer, 1 stomach, 1 other cancer
T49	IDC	II	+	+	-	+	*YES*		1 bilateral breast, 1 uterine cancer(abuela paterna)
T2	DCIS	_	+	+	-	+			2 breast, 1 liver cancer
T8	ILC	_	+	-	-	+			1 bilateral breast cancer
T13	DCIS	_	+	+	-	+			3 breast, 1 uterine, 2 stomach cancer
T22	IDC	II	+	+	-	+			2 breast, 2 ovarian, 1 lung cancer, 1 lymphoma
T28	DCIS	_	+	+	1+	+			3 breast cancer, 1 esophageal cancer
T30	ILC	_	+	+	1+	+			3 breast, 1 lymphoma
T31	IDC	I	+	+	-	+			3 breast, 1 prostate, 1 uterine cancer
T33	IDC	III	+	+	-	+			5 breast, 1 bilateral breast, 1 stomach, 1 pancreatic cancer
T34	IDC	II	+	+	-	+			5 breast, 1 bilateral breast, 1 stomach, 1 pancreatic cancer
T38	LCIS	_	+	+	-	+			4 breast (1 bilateral), 1 testicular, 1 other cancer
T47	IDC	II	+	+	-	+			2 breast, 1 prostate OR 2 breast, 1 stomach cancer
T48	IDC	III	+	-	-	+			3 breast cancer
T14	DCIS	_	+	+	2+	+			5 breast cancer
T51	IDC	II	+	+	ND	ND		*YES*	2 breast, 1 stomach cancer, 1 colon cancer, 1 myeloma
T50	IDC	III	+	-	1+	ND		*YES*	2 breast, 1 stomach cancer

*IDC* Invasive Ductal Carcinoma, *ILC* Invasive Lobular Carcinoma, *DCIS* Ductal Carcinoma *in situ*, *LCIS* Lobular Carcinoma *in situ*

*ND* Not determined

### Genomic losses and gains in BRCAX breast cancer tumors

Tables 2 and 3 show a list of losses and gains present in more than 10 % of BRCAX tumors including the most frequent alterations highlighted in bold. In each table, candidate “tumor suppressor genes” or “oncogenes” are indicated. The two most frequent genomic losses are present concomitantly in 9 BRCAX tumors (22.5 % in Table 2). It is relevant that 9 tumors have a deletion of two genes previously related to cancer progression such as *PLEKHO1* [26] a negative regulator of the mitogenic PI3K/AKT signaling pathway and *APH1A* [27] which loss of expression has been associated to poor survival in triple negative breast cancer patients [27]. Interestingly, a second group of tumors (15 % in Table 2) presented deletions at 9 regions simultaneously, all of them including several genes previously associated to cancer such as *PSMB8* [28], *HLA-DMB* [29], *SSBP1* [30] and *CADM1* [31].

**Table 2:** Genomic losses found in more than 10 % of BRCAX breast tumors

Chromosome	CytoBand	Start	Stop	Size bp	Percentage of BRCAX tumors (*N* = 40)	Candidate tumor suppressor genes	Other genes
1	p31.1	78765816	79106841	341,025	12.5		PTGFR, IFI44L, IFI44
	**q21.2**	**148392365**	**148504936**	**112,571**	**22.5**	**PLEKHO1, APH1A**	**ANP32E, CA14**
	q24.2	167969938	168079511	109,573	12.5		C1orf156, C1orf112
3	q25.1	152529936	152650652	120,716	12.5		MED12L,P2RY13, P2RY12, IGSF10
6	p21.32	32897974	32905723	7749	15.0		TAP2
	p21.32	32918832	32929682	10,850	17.5	PSMB8	TAP1
	p21.32	32932575	33057062	124,487	15.0	HLA-DMB	PSMB9, BRD2
	**p22.1**	**27200902**	**27210109**	**9207**	**20.0**		**HIST1H2BJ, HIST1H2AG**
7	q34	141051502	141137338	85,836	17.5	SSBP1	WEE2, TAS2R3, TAS2R4, TAS2R5
9	q32	116094655	116176804	82,149	15.0		COL27A1, ORM1, ORM2, AKNA
11	q23.3	114614479	116165823	1,551,344	15.0	CADM1	BUD13, ZPR1, APOA5
12	q13.2	54429832	54500555	70,723	12.5		GDF11, CIP29, ORMDL2
13	q21.1	52406170	52944596	538,426	12.5		OLFM4
14	q11.2	22424322	22467920	43,598	15.0		REM2, RBM23, PRMT5
15	q11.2	20477397	20599137	121,740	15.0	CYFIP1	NIPA2, NIPA1
16	q12.1	50773858	51032886	259,028	15.0		TOX3
17	**q25.1**	**68713135**	**68845671**	**132,536**	**20.0**		**COG1, FAM104A, C17orf80, CDC42EP4, SDK2**
20	**q12**	**39100100**	**39142168**	**42,068**	**22.5**		**TOP1**
	q12	39201874	39331155	129,281	12.5		PLCG1, ZHX3
22	q11.21	19683237	19692296	9059	12.5		LZTR1, THAP7

Most frequent losses in BRCAX tumors are highlighted in bold

**Table 3:** Genomic gains found in more than 10 % of BRCAX breast tumors

Chromosome	CytoBand	Start	Stop	Size bp	Percentage of BRCAX tumors (*N* = 40)	Candidate oncogenes	Other genes
1	**q21.1**	**143706304**	**143905470**	**199,166**	**15.0**	**PDE4DIP**	**SEC22B**
	q21.1	144219515	144279910	60,395	12.5	RBM8A	GNRHR2, PEX11B, ITGA10, ANKRD35
	q21.2	148240535	148367347	126,812	12.5	OTUD7B	VPS45
	**q21.2**	**148392365**	**148504936**	**112,571**	**17.5**		**PLEKHO1, APH1A, ANP32E, CA14**
	**q21.2**	**148519890**	**148564234**	**44,344**	**15.0**		**C1orf54, C1orf51, MRPS21, PRPF3**
	q32.1	201456918	201966787	509,869	12.5	BTG2	CHIT1, FMOD, ATP2B4
	q32.1	202269067	202358437	89,370	12.5		C1orf157, SOX13
	**q32.1**	**205037481**	**205260296**	**222,815**	**15.0**	**IL19, IL20, FAIM3**	**IL24, PIGR, FCAMR, C1orf116**
	q32.2	205762617	206263053	500,436	12.5	CD46, PLXNA2	CR1, CR1L, CD34
	q32.2	207826895	208566054	739,159	12.5	TRAF3IP3,LAMB3	G0S2, HSD11B1, C1orf74, IRF6, C1orf107, SYT14, SERTAD4
	q41	213425725	213768607	342,882	12.5		KCNK2
	q42.12	223406336	224419278	1,012,942	12.5	ENAH, LBR	DNAH14, SRP9, EPHX1, TMEM63A, LEFTY1, PYCR2, LEFTY2, C1orf55, H3F3A, ACBD3
	q42.13	225961050	226071970	110,920	12.5		JMJD4, SNAP47, MPN2
6	p21.33	31663820	31905687	241,867	12.5	CLIC1, CSNK2B	LST1, NCR3, AIF1, BAT2, BAT3, APOM, BAT4, C6orf47, LY6G5B, LY6G5C, BAT5, LY6G6F, LY6G6E, LY6G6D, LY6G6C, DDAH2, MSH5, C6orf27, VARS, LSM2, HSPA1A, HSPA1B
8	q22.1	98923270	99014727	91,457	12.5	LAPTM4B	MATN2
	q22.3	104310836	104453937	143,101	12.5	FZD6, CTHRC1	BAALC
	q23.1	107173263	107833235	659,972	12.5		OXR1
	q24.13	124926272	125341753	415,481	12.5		FER1L6
	q24.21	130632541	130857683	225,142	12.5		GSDMC
12	q13.2	54405492	54500555	95,063	12.5	CIP29	CD63, GDF11, ORMDL2
17	q12	34260921	34473439	212,518	12.5	RPL23, PLXDC1, LASP1	FBXO47
19	q13.33	55918797	56055048	136,251	12.5	KLK15, KLK3	CLEC11A, GPR32, ACPT, C19orf48, KLK1
	q13.42	60568830	60853735	284,905	12.5	IL11, UBE2S	TMEM190, RPL28, ZNF579, FIZ1, ZNF524, ZNF580, ZNF581, CCDC106
20	**q12**	**39100100**	**39358266**	**258,166**	**15.0**	**PLCG1, TOP1**	**PRO0628, ZHX3**

Most frequent gains in BRCAX tumors are highlighted in bold

The most frequent gains found in our BRCAX tumors (Table 3) have been previously observed to be amplified in breast cancer [7, 8, 12], and contain at least four genes of interest *PDE4DIP*/Myomegalin [32, 33], *IL19, IL20* [34–36] and *FAIM3* [37–39]. The gain of these regions is in agreement with the overexpression observed in breast tumors for all these genes. Specially, IL19 has been proposed as a prognostic marker in breast cancer, and its expression is correlated to advanced tumor stage, metastasis, and poor survival [34, 36]. In this way, targeting IL19 could become a good therapy for breast cancer patients.

### Specific genomic alterations in hereditary tumors from BRCA1 and BRCA2 mutation carriers

In order to find specific alterations for *BRCA1* and *BRCA2* mutated tumors, we filtered out all those present in BRCAX tumors. Table 4 shows the genomic losses and gains present only in *BRCA1* and *BRCA2* tumors, highlighting in bold the genes already associated to cancer. Our analysis showed that DNA samples from *BRCA1* and *BRCA2* tumors carry common alterations (3/7 tumors), which are mainly deletions. We admit that our sample of seven *BRCA1* and *2* germline mutated tumors is small, but we felt important to highlight recurrent genomic alterations, not present in BRCAX tumors, since this has not been described in previous studies. Interestingly, one of these genes, E2F6, acts as a repressor of BRCA1 transcription [40, 41]. The overexpression of this transcriptional repressor in breast tumors may be a relevant mechanism for BRCA1 silencing.

**Table 4:** Genomic deletions and gains shared by 2 or more germline mutated tumors

					BRCA2 mutated tumors				BRCA1 mutated tumors		
					Mutation 1		Mutation 2		Mutation 3	Mutation 4	
Chr region	Start	Stop	Size bp	Genes	T50	T5	T51	T44	T49	T24	T25
1q41	212228277	212570219	341942	PROX1, **SMYD2**		Loss		Loss			
2p25.1	11198066	11682912	484846	PQLC3, **ROCK2, E2F6, GREB1**				Gain		Gain	
2q33.1	197759971	197921182	161211	ANKRD44				Loss		Loss	
2q33.2	203984291	204102868	118577	ABI2, **RAPH1**	Loss	Loss				Loss	
4q32.3-4q33	167949877	170912917	2963040	SPOCK3, **ANXA10**, DDX60, PALLD, CBR4, **SH3RF1, NEK1**, CLCN3	Loss	Loss					
4q34.1-4q34.2	175832063	176792165	960102	GLRA3, **ADAM29**, GMP6A	Loss					Loss	
7p13	43308108	44125072	816964	**HECW1, STK17A**, BLVRA, MRPS24, **URG4**, UDE2D4, **DBNL**, PGAM2, **POLM,** AEBP1, POLD2		Loss		Loss			
11q12.1	58254147	58647789	393642	GLYAT, GLYATL2, GLYATL1	Loss	Loss					
17q21.2	38450905	38521318	70413	**BRCA1**				Loss	Loss	Loss	
17q23.2	57344164	57454012	109848	INTS2, MED13		Loss			Loss		
19q13.11	40168316	40221937	53621	GRAMD1A, SCN1B	Loss				Loss		
20q13.12	44813919	45159168	345249	EYA2	Loss			Loss	Loss		

In bold are highlighted cancer associated genes found in genomic losses and gains present only in BRCA1 and BRCA2 tumors

Interestingly, tumors with the same *BRCA2* mutation T5 and T50 have a common genomic profile (Table 4). This is in line with a previous study by Alvarez et al [14], where they show that tumors with the same recurrent mutation in *BRCA2* share similar alterations. One alteration in these tumors that caught our attention was the 3 Mb loss in chromosome 4, which comprise at least three genes relevant for tumor suppression: *NEK1, POSH* and *ANX10A* (Table 4) [42–44]. These genes participate either in DNA repair and checkpoint control, apoptosis or in the regulation of cell proliferation, adding other crucial targets for cancer progression besides BRCA1 and BRCA2 dependent DNA repair.

In addition to the specific alterations, we found an interesting deletion at 3p12 in three *BRCA2* mutated tumors involving the genes for ROBO receptors 1 and 2. These genes encode for receptors of the SLIT/ROBO pathway, demonstrated to promote tumor suppression in breast cancer cell lines by impairing AKT/PI3K signaling [45]. On the other hand, some BRCAX tumors present loss of *SLIT2* loci, a ROBO ligand. Both results together strongly suggest that the inactivation of this pathway is necessary for the progression of *BRCA2* and BRCAX tumors. In a previous work from our group [46] we found a high percentage of hereditary tumors with loss of SLIT2 protein expression related to the hypermethylation of its promoter. These findings support the relevance of the silencing of the SLIT/ROBO pathway for the progression of hereditary breast cancer.

### BRCA1 expression and genomic alterations in hereditary breast tumors

We have previously evaluated BRCA1 protein expression in these tumors through immunohistochemistry [17]. We found twenty four tumors with a negative expression of BRCA1 in the nucleus, two of them carrying a germline *BRCA1* mutation. Among the tumors with no *BRCA1* mutations and loss of BRCA1 expression, we found 67 % with *BRCA1* promoter hypermethylation [17]. In addition, specific analysis of the *BRCA1* probes of the array in this study (data not shown) revealed partial or total deletion of *BRCA1* in 7 BRCA1 not expressing tumors (29 %). Since *BRCA1* is a relevant driver in breast cancer we analyzed gains and losses in these tumors to correlate the absence of BRCA1 protein to specific genomic alterations. On this respect, we found several recurrent deletions private for BRCA1 not expressing tumors: 1p36.13, 8p22, 9q32, 11q14.1, 11q23.3, 13q12.13, 15q22.33, 17p12, previously described in hereditary breast cancer tumors [8, 14, 15, 22]. Two of these regions, 9q32 and 13q12, have been described also for *BRCA1* germline mutated tumors [12, 13]. In relation to this study, 8p22 region with at least six candidate tumor suppressor genes, was found lost in 4/24 BRCA1 not expressing tumors. Downregulation of four of these genes (*TUSC3, DLC1, ZDHHC2* and *MTUS1*) have been described associated to invasiveness and metastasis [47–50].

On the other hand a 3.6 Mbp gain in chromosome 12q21.1, including oncogenes *LGR5* (leucine-rich repeat containing G protein-coupled receptor 5) and *RAB21* (RAB21, member RAS oncogene family), was the most frequent gain found in BRCA1 not expressing tumors. Interestingly, in addition to the 4 genes described before, RAB21 has also been implicated in the invasiveness and metastasis of breast cancer cells in vitro [51].

### Clustering analysis revealed four major groups of hereditary tumors

**Fig. 2:**
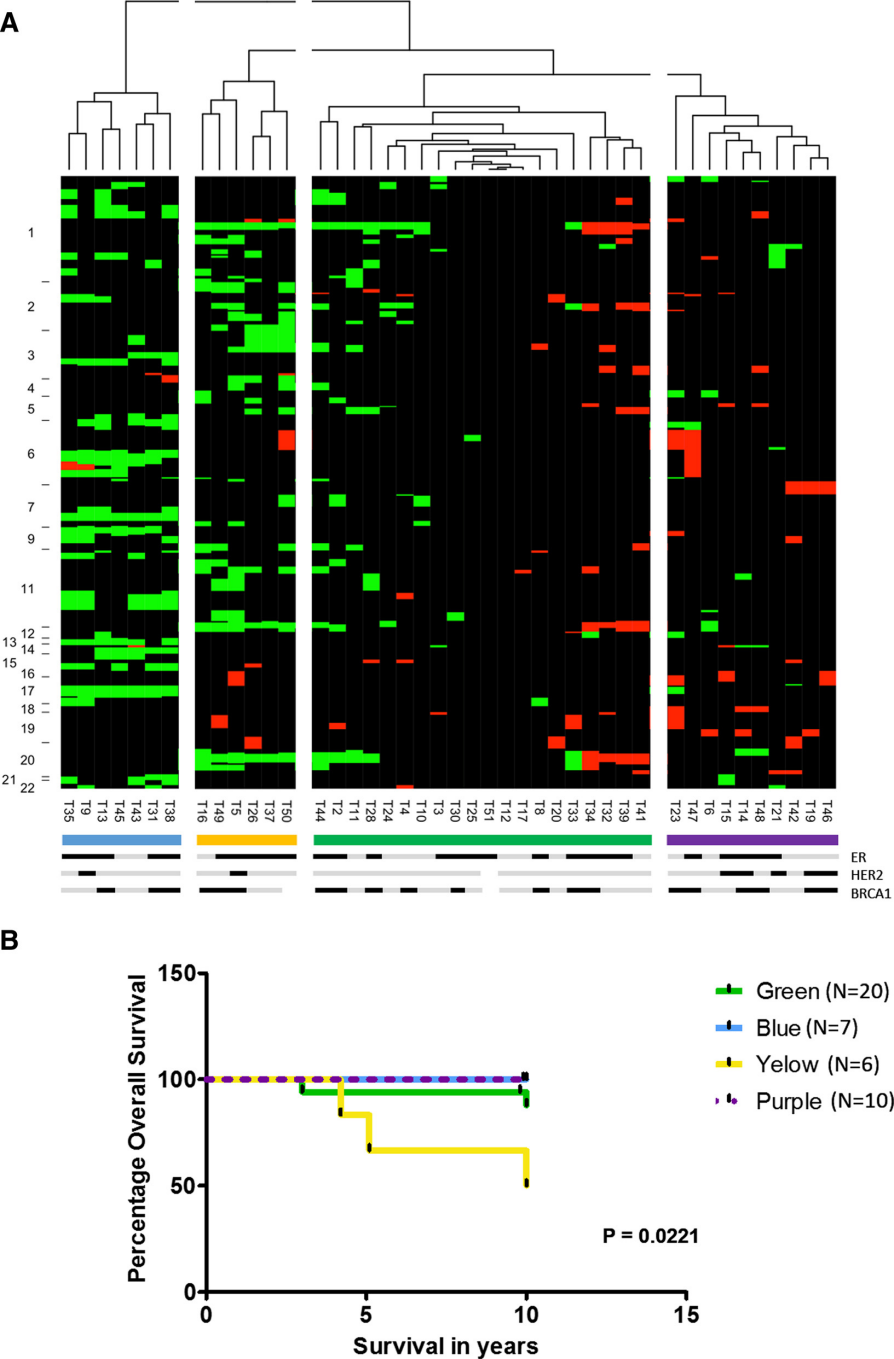
Cluster analysis of hereditary tumors. **a** Unsupervised hierarchical clustering for hereditary breast tumors. T1, T36, T22 and T29 were removed from the cluster as considered outliers. Numbers in the Y- axis correspond to each chromosome and the marks in the Y-axis are the limits between chromosomes. Green boxes: losses, Red boxes: gains, Black boxes: no change. Four groups were identified and labeled with Blue, Yellow, Green and Purple lines under the picture. In addition, ER, HER2 and BRCA1 expression status is indicated below as follows: black: positive, grey: negative, white: no information. **b** Overall survival of the 4 clusters determined by Log-rank (Mantel-Cox) Test, *p* < 0.05. Tumors from each cluster are represented with a respective color line

In order to identify the major rearrangements that characterize different hereditary tumors we clustered our samples into four groups using array CGH data. Figure 2a shows four major groups of tumors characterized by the type of alteration (loss or gain), the amount of alterations, and/or their size. The Blue and Yellow clusters carry mainly deletions that clearly distinguished these tumors. Most of these alterations are shown in Table 2, and include genes associated to immune response (Blue) and cell cycle regulation (Yellow). The Purple cluster tumors carry mainly gains involving genes associated to migration, invasion and metastasis in breast and other cancers. Finally, the Green cluster is a more heterogeneous group, characterized by tumors carrying a significant lower number of gains and/or losses compared to the other clusters (Student *T*-test, *p* values = Blue vs Green 0.00029, Yellow vs Green 0.003106, Purple vs Green 0.004513).

Interestingly, regarding receptor status and tumor clustering, five of the seven (71.4 %) HER2 positive tumors were grouped in the Purple cluster (Fig. 2a), and none were contained in the large Green cluster. ER positive tumors instead were distributed equitably along the four groups, as well as BRCA1 not expressing tumors.

We performed overall survival analysis using Log-rank (Mantel-Cox) Test considering data available from all patients (Fig. 2b). Analysis of the four groups revealed a significant poor survival at 10 years after surgery, for patients carrying tumors in the yellow cluster (*p* value = 0.0221).

### Gene ontology enrichment

Analysis with GOrilla [24] and DAVID [25] showed different cellular processes affected in different groups of tumors (Table 5). In *BRCA1/2* mutated and BRCA1 not expressing tumors, both having an impaired DNA double strand break repair, we found common cellular processes affected such as apoptosis, chromatin organization/DNA packaging and transcription. These results suggest that breast cancer tumors with non-functional BRCA1, due to any of the mentioned factors, share the impairment of the same cellular processes caused by BRCA1 absence or deficiency.

**Table 5:** Gene ontology enrichment in different groups of hereditary tumors

Tumor groups	Enriched Gene Ontologies
*BRCA1/2 mutated*	Regulation of cytoskeleton organization, Negative regulation of mammary epithelial cell proliferation, Protein modification process, Apoptosis, Cell cycle regulation, RNA transcription and processing, DNA damage repair, DNA packaging
*BRCA1 not expressing*	Alpha aminoacid metabolic/biosynthetic processes, Protein citrullination and Citrulline metabolism, Proteolysis, Transcription, Chromosome segregation and chromatin organization, Apoptosis
*BRCA1 expressing*	No enrichment was found
*Blue cluster*	Antigen processing and presentation (13 GO Terms), Intracellular transport
*Yellow cluster*	Cytokine signaling, Collagen metabolic processes and Extracellular matrix organization
*Purple cluster*	Calcium-independent cell-cell adhesion
*Green cluster*	No enrichment was found

Considering the four clusters, distinct processes were identified indicating different tumor progression programs (Table 5). No significant enrichment was found within the green cluster. As previously mentioned, the Yellow cluster showed a poor survival compared to the rest of the tumor clusters. Within the enriched processes affected in these tumors we found two relevant genes, *DARC* (Duffy antigen receptor for chemokines) and *DAG1* (α-Dystroglycan). The loss of expression of these genes has been associated with poor survival of breast cancer patients [52–54]. This association is probably due to the aggressiveness and metastatic potential that tumor cells acquire in the absence of the function of these genes.

## Discussion

We analyzed through array CGH the genomic profile of 47 biopsies, from hereditary breast cancer patients, 40 from *BRCAX* patients, 3 from *BRCA1* and 4 from *BRCA2* mutation carriers (Table 1). To our knowledge this is the first study on genomic alterations, gene functions and molecular pathways involved in hereditary breast cancer tumors, in a Latin American population. The relevance of this study is based on the influence of Genetics and Environment as two key factors in cancer progression.

We found several chromosomal alterations with low frequency in hereditary breast cancer tumors, revealing high inter-tumor heterogeneity at the genomic level. As stated in results, the higher frequency of deletions or gains was 22.5 % among BRCAX tumors. Within the identified alterations in BRCAX tumors, several regions have been previously identified in similar studies for non-BRCA1/2 familial cancer, such as loss in 11q and 16q, and gains in 1q and 8q [14, 55].

In relation to tumors with BRCA1/2 germline mutations, frequency of recurrent alterations rises to 75 % within *BRCA2* tumors, and 66 % within *BRCA1* tumors. In addition to the most recurrent alterations, our work describes the presence of genomic alterations present only in the BRCA1/2 mutated tumors. Previous reports have described common alterations in *BRCA1* and *BRCA2* tumors [8, 13, 14, 55], that are also present in sporadic or familial BRCAX tumors, although in a lower frequency. Within the regions described in the literature, loss of 4q, 3p, 12q in *BRCA1* tumors, and loss of 11q and 13q for *BRCA2* are recurrent. In our tumors all the previous alterations were found, being loss of 4q and 11q present only in our BRCA mutated tumors. Among the regions described as altered for *BRCA1*/2 tumors in our study we found several genes that have been previously associated with relevant cellular processes such as DNA repair, cell growth and apoptosis.

Clustering of hereditary tumors using genomic alterations revealed that the tumors of the Yellow cluster have significant poor overall survival compared to the rest of the groups (Fig. 2b). In this relation, *DARC* and *DAG1* genes, contained in the frequent genomic losses in the Yellow cluster, have been previously associated to poor survival. DAG1 encodes α-Dystroglycan, a highly relevant glycoprotein that binds to laminin maintaining the correct organization of epithelial tissues [56]. On the other hand, *DARC* as a chemokine receptor has a major role in inflammation, a process commonly present during invasion of tumor cells. In this sense, the loss of expression of these two genes associated to a poor prognosis, maybe due to a higher incidence of metastasis in these patients [52–54]. In addition, as described in results the Yellow cluster present frequent a loss of *PLEKHO1* and *GDF11* genes, regulators of PI3K/AKT and EGF signaling, respectively. These two pathways have been extensively cited as highly activated in triple negative breast cancer tumors, which are well known for having a poor overall survival with respect to other breast cancer subtypes [57]. The contribution of the activation of PI3K/AKT and EGF pathways to poor survival has been related to the lower response and/or resistance to chemotherapy observed in patients [58, 59]. Finally, we also found loss of *CD63* (member of the tetraspanin family), an event previously associated to advanced stages of melanoma [60]. The involvement of *CD63* in cancer metastasis and its loss in tumors described in this study, is in concordance with a poorer overall survival of patients in the yellow cluster. The Blue cluster have also interesting features, since losses found in this group involve genes related to the processing and presentation of immunogenic peptides, which are frequently downregulated in different types of cancer (Cluster analysis section in Results). Downregulation of these genes affect peptide characteristics and their transport to the endoplasmic reticulum for its binding by MHC class I proteins. In this regard, tumors presenting these deletions will have a possibility for treatment with specific immunotherapy.

We found significant differences in the number of alterations between clusters, having the Green cluster the lower instability compared with Blue, Yellow and Purple clusters. A previous work by Stefansson et al [13] analyzed 29 tumors defined as “with BRCA alterations” (*BRCA1/2* mutation or *BRCA1* hypermethylation/loss of expression) compared to 38 sporadic tumors without any BRCA alteration. These authors described 4 clusters of tumors, three of which present a high instability, like in our study. Among those three clusters, two were enriched in BRCA altered tumors presenting mainly big size losses. This is consistent with our results, since the Yellow cluster (6 tumors) having high genomic instability and characterized mainly by losses, is enriched in *BRCA1* and *BRCA2* mutated tumors (3/6 tumors). In addition to this concordance with Stefansson’s results, regarding hereditary BRCA1 or 2 deficient tumors, we added to the knowledge the fact that this instable BRCA-enriched cluster has a poor overall survival, as mentioned in the previous paragraph. Our results in hereditary tumors are also consistent with Fridlyand et al [11], who described three groups of sporadic breast cancer tumors with differences in CNA number and type, and with survival.

Although we found in our tumors, genomic alterations previously described in the literature, these are present in a low proportion of tumors. In addition, it comes to our attention that tumors of the Green cluster, gathering almost half of our hereditary tumors, have a low number of alterations. Latin American populations, like the one in this study, constitute an admixture of Spanish and Amerindian individuals, being genetically different from breast cancer cases frequently analyzed in similar studies. These ethnic differences in conjunction with environmental factors may lead into differences in the molecular mechanisms of cancer progression among populations.

In our study, we included different pathological subtypes such as ductal and lobular *in situ* and invasive carcinomas. According to our results, these carcinomas are distributed across all clusters, indicating that *in situ* diseases are as heterogeneous as, and behave similar to, the invasive tumors.

*BRCA1* silencing in sporadic and hereditary tumors have been described in the last years to be a relevant mechanism associated to breast cancer progression in patients with no germline mutation [16, 17]. In our study, small groups of BRCA1 not expressing tumors share common genomic alterations though the majority of tumors do not have the same genes affected. Nonetheless, the relevant cellular processes highlighted for these tumors revealed that the affected genes, although different, involve the same molecular pathways. This observation is in agreement with previous reports describing core affected pathways in pancreatic cancer [61, 62]. In addition, we identified genomic alterations and cellular processes shared by *BRCA1* mutated and BRCA1 not expressing tumors. This is in line with the fact that some tumors, lacking germline mutations in *BRCA1* show a BRCAness phenotype, implying that they could have a cancer progression program similar to *BRCA1* mutated tumors.

The results obtained for BRCA1 not expressing tumors suggest a more relevant contribution of BRCA1 functional deficiency to the general genomic instability of the tumors than to the development of specific alterations. As observed, none of the tumor clusters are characterized by a particular BRCA1 expression status, but they do carry common alterations (Fig. 2a). This evidence may reflect that the consequences of BRCA1 functional deficiency depend on the genetic background of the tumors, the mechanism of inactivation, or the moment at which this event occurs. Moreover, it is necessary to determine whether other alterations of BRCA1 function, such as cytoplasmic retention, somatic mutations or post-translational regulation by miRNAs may contribute to the particular genomic profiles observed in each cluster.

Array CGH have been used in recent years to get relevant information for clinical trials. Two prospective trials, SAFIR01 and MOSCATO, intend to destine patients to different targeted therapies depending on genomic gains and somatic mutations affecting relevant targets for therapy. In these studies, amplifications of low recurrence involving genes such as *EGFR, FGFR* and *FGF* ligands, *AKT, PIK3CA* and *IGF1R* are suitable markers for moderate or good antitumor response (stable disease or remission) to specific inhibitor for these pathways. In our study (data not shown), amplification of *AKT, PIK3CA* and *FGF* receptors and ligands were observed in BRCA1 not expressing tumors, opening a new therapeutic opportunity for tumors with a BRCAness phenotype. In this relation, it has already been demonstrated in triple negative breast cancer cell lines, that combining PI3K and EGFR inhibitors produces a better response than each inhibitor alone [63] becoming a promising strategy for BRCAness tumors treatment. In addition, a group of our tumors (Yellow cluster) exhibit deletions of *PLEKHO1* and *GDF11*, which products regulate PI3K and EGF signaling pathways. Patients carrying this type of tumors, showing a poor overall survival, could be good candidates for the combined therapy mentioned before. These therapies may bring an alternative treatment to patients carrying BRCAness tumors, or could be used in combination with PARP inhibitors.

## Conclusion

Our results support the fact that BRCA1 expression in tumors should be used as a marker for BRCAness and for selection of these patients for synthetic lethality approaches such as treatment with PARP inhibitors. In addition, the identification of specific alterations in breast tumors associated with poor survival, immune response or with a BRCAness phenotype will allow the use of a more personalized treatment in these patients.

## Abbreviations

aCGH: Array based Comparative Genomic Hybridization
ADM-1: Aberration Detection Method-1
ASCO: American Society of Clinical Oncology
BRCAX: Hereditary breast cancer without BRCA1 or BRCA2 germline mutations
CAP: College of American Pathologists
CNV: Copy Number Variation
ER: Estrogen Receptor
FFPE: **F**ormalin-Fixed Paraffin Embedded
H&E: Hematoxylin-Eosin staining
